# Induction and Characteristics of Callus Cultures of the Medicinal Plant *Tussilago farfara* L.

**DOI:** 10.3390/plants13213080

**Published:** 2024-11-01

**Authors:** Monika Bojko, Magdalena Kędra, Agata Adamska, Zuzanna Jakubowska, Monika Tuleja, Beata Myśliwa-Kurdziel

**Affiliations:** 1 Department of Plant Physiology and Biochemistry, Faculty of Biochemistry, Biophysics and Biotechnology, Jagiellonian University in Kraków, Gronostajowa 7, 30-387 Kraków, Poland; m.bojko@uj.edu.pl (M.B.); magdalena.kedra@uj.edu.pl (M.K.); agata.adamska@student.uj.edu.pl (A.A.); zuzanna.jakubowska@doctoral.uj.edu.pl (Z.J.); 2 Doctoral School of Exact and Natural Sciences, Jagiellonian University in Kraków, Prof. S. Łojasiewicza 11, 30-348 Kraków, Poland; 3 Department of Plant Cytology and Embryology, Institute of Botany, Faculty of Biology, Jagiellonian University in Kraków, Gronostajowa 9, 30-387 Kraków, Poland

**Keywords:** carotenoids, chlorophyll, coltsfoot, fluorescence, histology, organogenesis

## Abstract

*Tussilago farfara* L. is a traditional medicinal plant valued for its potentially health-promoting metabolites. Its herbal raw material has been recognized and used since ancient times and continues to be widely used in traditional medicine. Introducing this plant species to in vitro cultivation is a challenging task, but once the protocol is developed, such cultures can provide an abundant and inexhaustible source of plant material. In this study, we report the successful induction and growth of vigorous *T. farfara* callus in vitro. Callus induction was achieved on MS solid media with the combination of indole-3-acetic acid (3 mg/L) and benzyl aminopurine (2 mg/L) in darkness, whereas it appeared inefficient under light conditions and in suspension culture. We present a detailed description of callus growth kinetics, morphological analysis, photosynthetic activity, and biochemical parameters (including protein content and photosynthetic pigments) supported by histological studies. Furthermore, we observed the potential for organogenesis and somatic embryogenesis. This method for the in vitro propagation of *T. farfara,* along with callus culture maintenance, offers a wide range of applications in pharmacy for the production of valuable metabolites. Moreover, it could benefit the environment by reducing the depletion of natural populations of this species and may serve as an alternative strategy for species conservation in light of global warming.

## 1. Introduction

**Figure 1:**
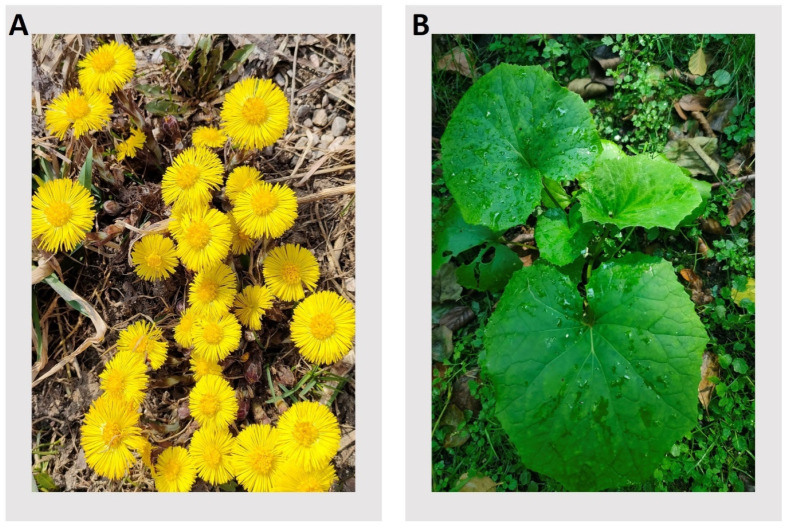
*Tussilago farfara* L. in its natural habitat (meadows near the Campus of the 600th Anniversary of the Jagiellonian University in Kraków (50°01′44,396″ N 19°54′17,496″ E; Poland). (**A**): Yellow inflorescences (capitula) visible in early spring, developing before the leaves, alongside thin, underground branched rhizomes. (**B**): Large, round, heart-shaped leaves with radial veins and crinkly, slightly toothed edges, documented in summer.

*Tussilago farfara* L. (coltsfoot; Figure 1) is a perennial species of plant from the *Asteraceae* family, a representative of the monotypic genus *Tussilago,* which grows primarily in the temperate biome. Its native range includes Europe, a large part of Asia (excluding its southern and eastern ends), and northern Africa. It is currently distributed in up to 46 countries around the world. As an introduced species, it has spread, among others, to North America, India, and the Far East [1]. In Poland, *T. farfara* is a common species, typically found on clay soils. It prefers stony places, scree, riverside gravel pits, landslides, roadside slopes, the precipitous banks of rivers and streams, coal dumps, and burnt areas. However, it should not be collected in areas where the soil is polluted by industrial waste [2]. In agricultural crops, *T. farfara* is considered a weed. In the classification of plant communities, this species is characteristic of the association *Senecioni-Tussilaginetum* [3]. In introduced areas, coltsfoot can quickly form dense stands that aggressively invade well-established farmland, and it is considered a naturalized weed in parts of North America [4].

Coltsfoot is a herbaceous plant that has long been used in folk medicine. The traditional use of coltsfoot is mainly associated with respiratory health. It has been used to relieve some disorders of this system, such as coughs, bronchitis, and asthma. Its potential anti-inflammatory properties also seem interesting. In folk medicine, coltsfoot was commonly prepared as an infusion or decoction of dried leaves or flowers in water. Today, coltsfoot continues to attract attention from researchers investigating the potential healing properties of medicinal plant extracts. It is already known that the leaves and buds of this species are rich in chemicals, including sesquiterpenes, phenolic acids, flavonoids, alkaloids, and others [4,5,6]. Further research is required to elucidate coltsfoot’s secondary metabolites and to develop protocols for the extraction and purification of interesting compounds. Additionally, more studies are needed to elucidate the specific mechanisms of action and to determine the safety and effectiveness of coltsfoot in medical applications. Callus culture plays a key role in plant research. It serves as a model system for studying cellular processes, genetic variability, and the effects of various plant growth regulators and stress conditions on plant cells. A major advantage of this model is the controlled environment, which allows observation of cellular processes without the influence of external factors. Callus culture is also crucial for the conservation of endangered plant species, particularly those endangered today, due to rapidly changing climatic conditions [7]. Additionally, it aids in the development of genetically modified plants to improve agricultural performance and in modifying secondary metabolism to produce desired metabolites. Currently, biotechnology is a field in which callus cultures are successfully utilized for producing various primary and secondary metabolites, and scaling up the production of bioactive compounds for pharmaceutical, cosmetic, and food applications, as well as phytoremediation [8].

According to the definition, a callus is a mass of unorganized, diverse parenchymatous cells, among which there may be totipotent cells capable of regenerating the entire plant organism by organogenesis and/or somatic embryogenesis [9]. This ability facilitates the amplification of limited plant material and provides tools for genetic cell transformation, which are not only much more rapid than conventional breeding but also give rise to novel genes and genotypes [10]. It is commonly known that calli cultures are unstable and characterized by high genetic variability. This feature can be a disadvantage but can be converted into an advantage when such genetically modified callus cultures can be a source of novel bioactive secondary metabolites [11] and can lead to the generation of plants with improved resistance to salt, drought, diseases, and pests [7].

Considering all the advantages and wide applications of callus cultures, as well as the specificity of the species with a focus on its medicinal properties and the lack of data in the literature, the development of new protocols and a thorough analysis of cultures still seems necessary. Our research aimed to develop a protocol for inducing callus from *T. farfara* leaves and establishing callus cultures. Our primary objective was to monitor and evaluate callus growth kinetics, morphology, photosynthetic activity, and biochemical parameters (including protein content and photosynthetic pigments). Next, we aimed to perform a histological analysis. In addition to that, we intended to examine the potential for organogenesis and somatic embryogenesis. It should be emphasized that the studies were conducted on plant material already maintained in in vitro culture, which provides a basis for confirming the wide possibility of obtaining material even from secondary in vitro cultures.

## 2. Results and Discussion

**Figure 2:**
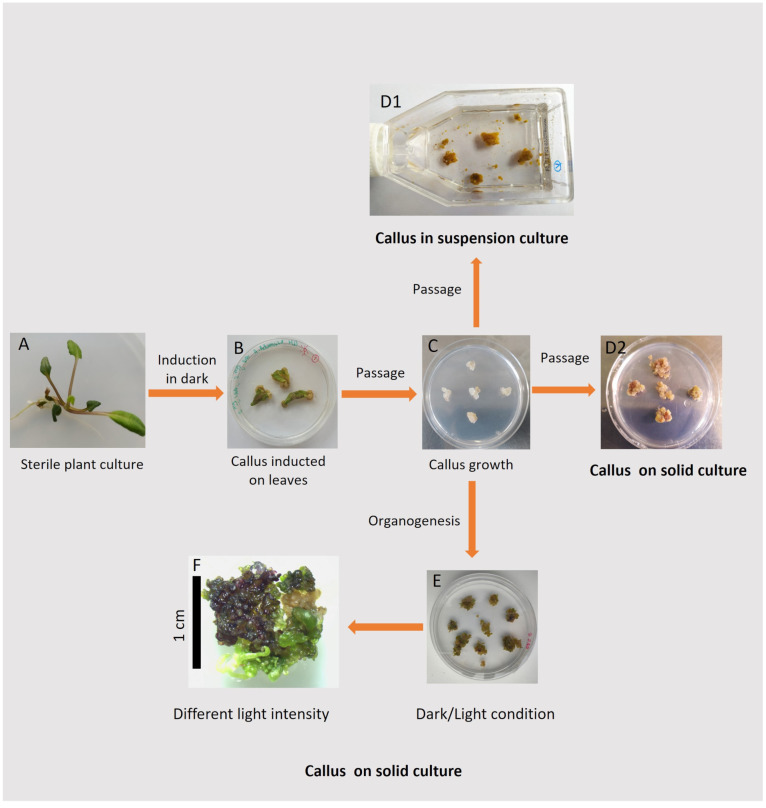
Schematic overview of the experiment, including illustrative examples of the results. Leaf explants were taken from a *T. farfara* plant, regenerated from a callus and grown in vitro (**A**), and used for the induction of a callus (**B**); A callus, separated from the leaf explants, is subcultured on solid medium (**C**); The callus is transferred to a suspension culture (**D1**) and on solid media (**D2**), with the addition of various hormones and grown in darkness. Finally, after 42 days of growth in darkness on solid media and in suspension culture, the callus tissue was taken for biochemical analyses (**D1**,**D2**). Additionally, stabilized callus cultures (**C**) were subcultured and grown under dark/light conditions and used for histochemical study (**E**); morphogenic callus from darkness (**C**), transferred to various light conditions (**F**).

The experiment encompassed the induction of a callus on leaf explants taken from a *T. farfara* plant that had already been regenerated from a callus. The newly induced callus was then grown under various conditions to determine the optimal growth conditions for callus development. The biochemical and histological characteristics of the callus, together with an analysis of its photosynthetic activity, were measured at various stages of the experiment. The overall experimental scheme is shown in Figure 2, which serves as a reference for describing the individual experimental steps and analyses detailed in the following subsections.

### 2.1. Callus Induction

**Figure 3:**
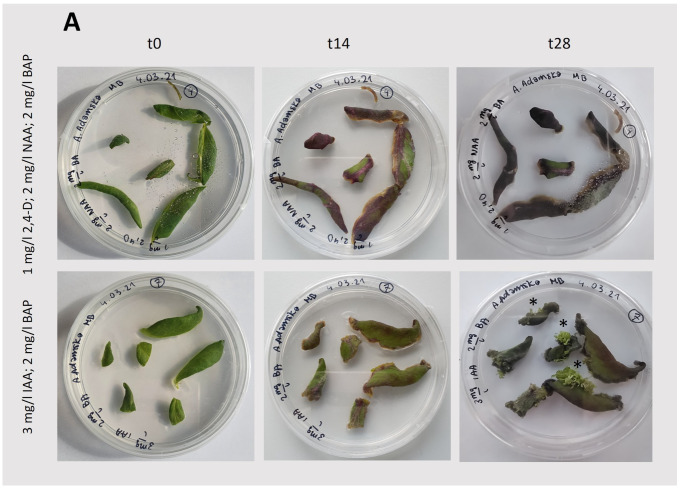

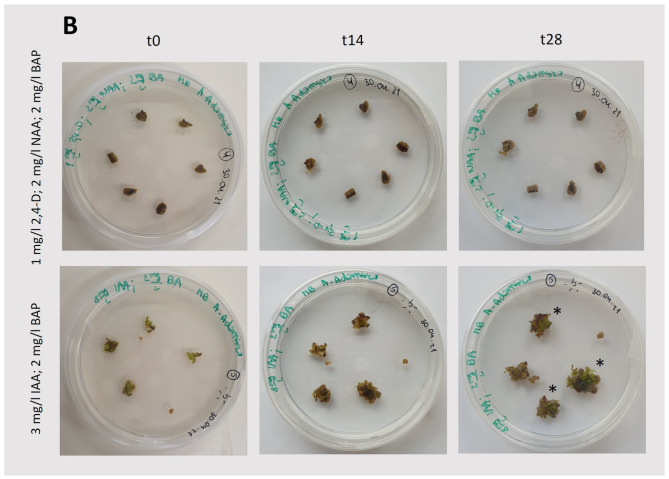
Induction and growth of the callus under light conditions (75 μmol photons m^−2^ s ^−1^; 14/10 (light/dark) photoperiod). (**A**): Callus induction from the leaf blade (Figure 2B). (**B**): Callus growth after initiation; t0—the day of callus passage, the starting point of observation; t14 and t28—observations after 14 and 28 days, respectively. In the case of the oldest cultures (t28), exemplary shoots are indicated by stars. The composition of the medium is specified in the figure.

**Figure 4:**
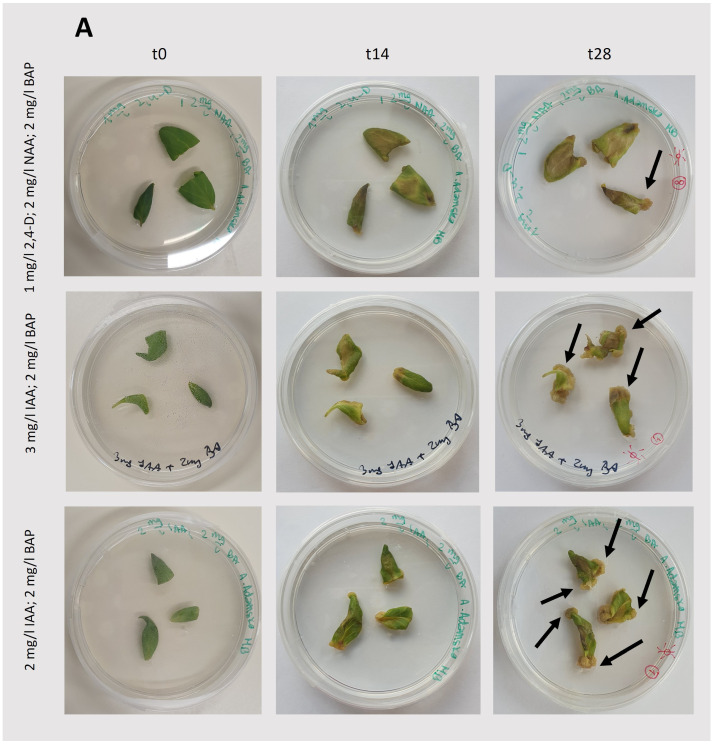

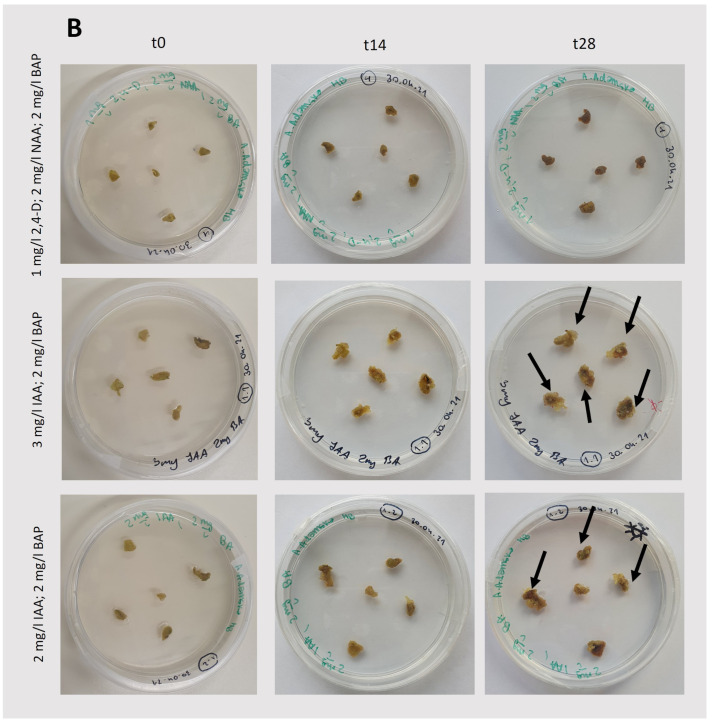
Induction and growth of the callus under dark conditions. (**A**): Callus induction from the leaf blade (Figure 2B). (**B**): Callus growth after initiation; t0—the day of callus passage, the starting point of observation; t14 and t28—observations after 14 and 28 days, respectively. In the case of the oldest cultures (t28), exemplary callus is pointed by arrows. The composition of the medium is specified in the figure.

For callus induction, leaf blades were taken from a *T. farfara* plant that had already been regenerated from a callus and cultivated in vitro (Figure 2A). The induction process was performed using different concentrations and types of hormones [12,13] under light and dark conditions (Figure 2B). The results of the tested conditions that ensured callus induction are summarized in Table 1, and representative photographs are shown in Figure 3 and Figure 4.

In light-grown cultures, the successful induction and growth of the callus were observed for each set of phytohormones tested (Figure 3; Table 1). However, the growth of this tissue immediately after initiation and after the first passage, still together with leaf fragments (Figure 2B), was quite limited. Furthermore, the use of indole-3-acetic acid (IAA; 3 mg/L) with benzyl aminopurine (BAP; 2 mg/L) under light conditions resulted in intensive bud differentiation and rhyzogenesis (Figure 3B), suggesting that these conditions are not suitable for the cultivation of a *Tussilago* callus. Similar to light-grown cultures, initiation and growth of the callus under dark conditions were observed in the presence of the tested phytohormones (Figure 4; Table 1). The largest callus amounts were obtained with the combination of IAA (3 mg/L) and BAP (2 mg/L). A combination of IAA (2 mg/L) and BAP (2 mg/L) also efficiently induced callus formation, although it was slightly less effective than the higher concentration of IAA.

Based on the analysis of callus growth after initiation and subsequent passages performed in the presence of various hormones, with or without light, the combination of IAA (3 mg/L) and BAP (2 mg/L) appeared to be the most effective condition for *T. farfara* callus growth. Similar results were observed in a study on the effects of various auxins and kinetins on callus induction on *Arachis hypogaea* cotyledons [12], where the most effective set of hormones inducing initiation and the highest increase in callus mass was IAA (3 mg/L) with a slightly lower concentration of BAP (1 mg/L) than in our study. However, the hormone combination that was previously identified as optimal for *T. farfara* callus induction (MS with 2,4-D; 2 mg/L and BAP; 3 mg/L) [13] induced the callus very poorly in our experiments and only under dark conditions (Table 1).

### 2.2. Analysis of Callus Growth Kinetics

**Figure 5:**
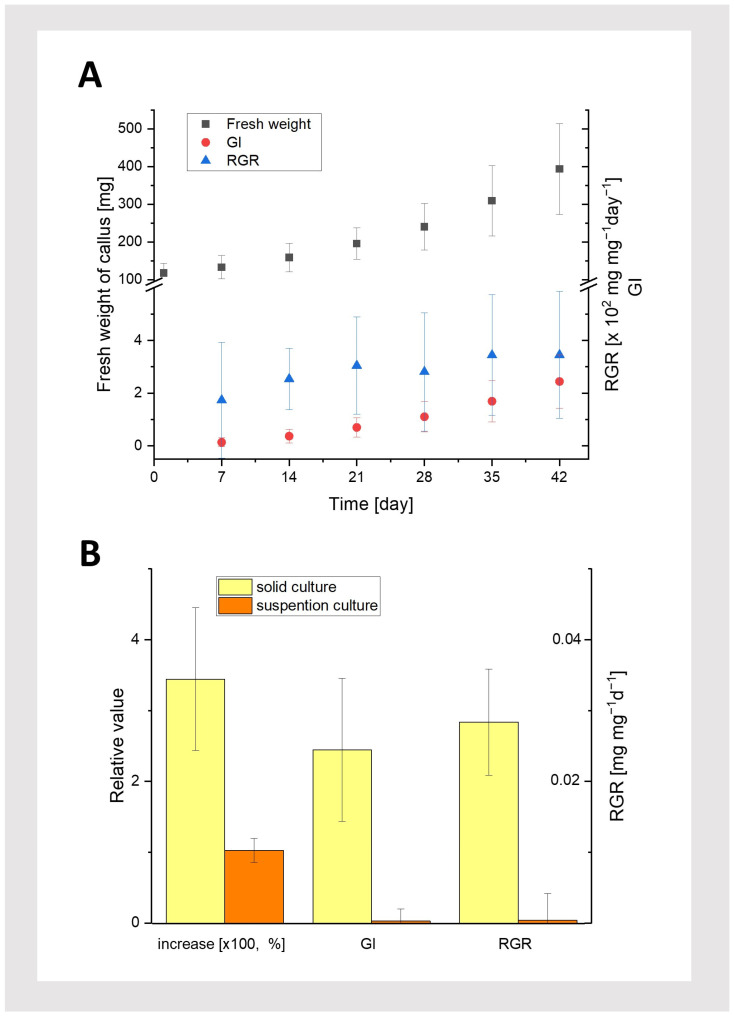
Callus growth characteristics. (**A**): Growth kinetics (fresh weight), Growth Index (GI), and Relative Growth Rate (RGR; harvest interval: 7 days) of the callus grown under dark conditions on solid media (MS, 3 mg/L IAA; 2 mg/L BAP, 0.6% agar). (**B**): Increase in fresh weight, Growth Index (GI), and Relative Growth Rate (RGR) for the callus from solid and suspension cultures (MS, 3 mg/L IAA; 2 mg/L BAP) under dark conditions, measured on the 42nd day of culture. Average values and standard deviations are shown for n = 25 callus fragments.

Callus growth kinetics were examined for cultures grown in the dark with the selected hormone combination (3 mg/L IAA and 2 mg/L BAP), i.e., under conditions where the callus grew most efficiently (Figure 2C). We compared callus growth in both solid and suspension cultures (Figure 2(D1,D2)). Growth kinetics were analyzed by measuring the increase in fresh weight and parameters such as the Growth Index (GI; [14]) and Relative Growth Rate (RGR; [15,16]). The results (fresh weight and GI) indicate intense callus growth on solid medium throughout the entire culture period under these conditions (Figure 5A). Moreover, the RGR parameter was almost constant, suggesting that the average weight increase per day remained steady. Additionally, the obtained values of the calculated RGR are similar to the rate for callus cultures of other species and confirm the growth phase of the *T. farfara* callus culture [15,16]. The callus tissue was a slightly darker yellow, similar in color to the initial tissue. These findings confirm that the culture parameters were appropriately selected, allowing for the production of a large quantity of vigorous callus material.

In contrast, the callus grown in liquid medium showed only a slight increase in tissue fresh weight. After 42 days of observation, the callus had turned brown and was significantly darker than the initial (passaged) tissue lumps at the beginning of the experiment. Furthermore, a comparison of tissue growth parameters on the 42nd day of both cultures (solid and suspension) revealed that tissue growth in liquid medium was minimal compared to the substantial growth observed on solid media (Figure 5B). Suspension cultures of pharmaceutically important plant cells are quite popular because they enable, for example, the controlled production and extraction of various secondary metabolites of pharmaceutical value in the culture medium. Although the suspension culture of callus cells has been used in many plant species [17], it turned out to be ineffective for *T. farfara* in our study. These results suggest that the liquid medium, under our experimental conditions, was unfavorable for *T. farfara* callus growth.

**Figure 6:**
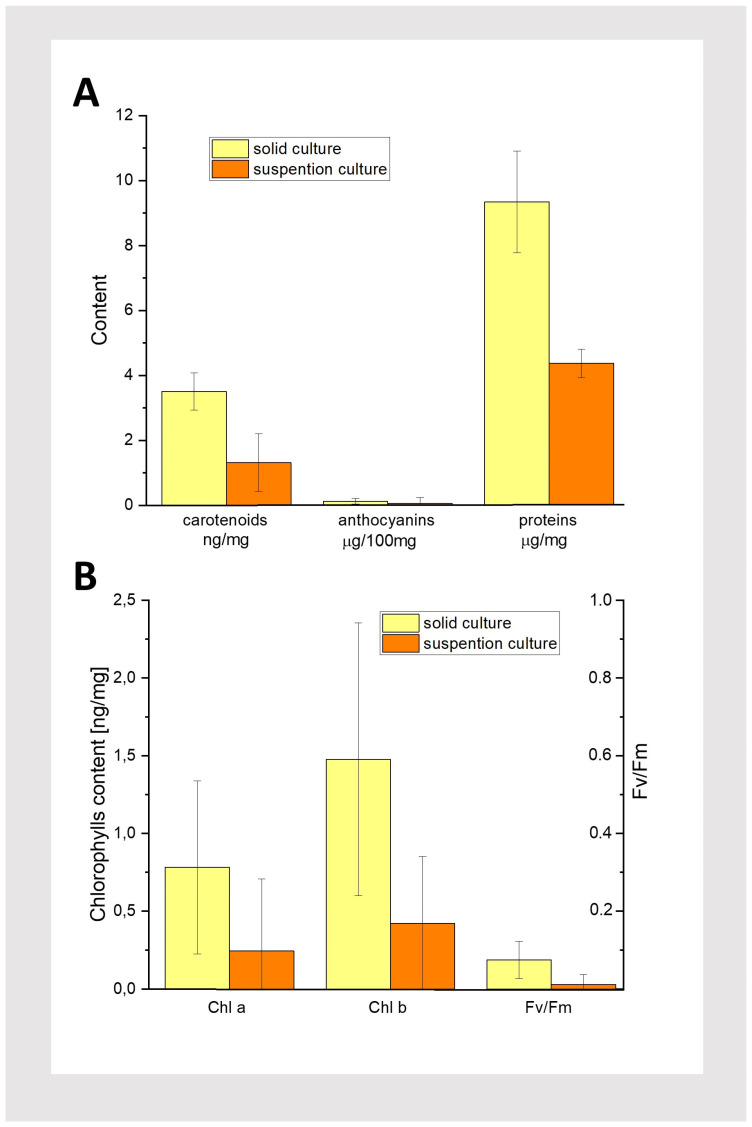
Results of biochemical tests performed on dark-grown 42-day-old solid and suspension cultures of *T. farfara*. (**A**): Content of carotenoids, anthocyanins, and proteins. (**B**): Content of chlorophylls and F_V_/F_M_; n = 3, n represents individual pieces of callus.

To gain more insight into the differences between solid and liquid medium cultures, we performed a series of biochemical tests after 42 days of cultivation, i.e., corresponding to the end of the liquid culture (Figure 2(D1,D2)). We focused on measuring the protein and pigment content, as well as F_V_/F_M_ representing the maximum quantum yield of photosystem II (PSII) [18]. The amounts of proteins, anthocyanins, and carotenoids in callus tissue are shown in Figure 6A. Proteins were found in tissues grown on both media. In solid cultures, their amount was comparable to that measured in intensively growing callus obtained from other plant species [19,20,21]. In contrast, in suspension culture, the protein concentration was over 50% lower than in cultures on solid medium when calculated on a fresh weight basis. The obtained results from the biochemical analysis confirm that solid cultures are more favorable than suspension cultures for *T. farfara* callus.

Trace amounts of anthocyanins were found in the analyzed callus (Figure 6A), even though the tissues were cultured in the dark. It has been shown that anthocyanin accumulation in the callus is typically stimulated by light [22]. Small amounts of photosynthetic pigments, such as carotenoids and chlorophylls, were also found in the callus from both solid and suspension cultures. Notably, the highest concentration was measured for carotenoids in solid cultures, at approximately 3.5 µg/mg of tissue, followed by Chl b at about half that amount, and the lowest concentration was found for Chl a. This explains the yellowish color of the callus observed in solid cultures. Interestingly, in both types of culture, comparable amounts of Chl a and Chl b were detected, with a slight excess of Chl b. Despite the presence of these trace amounts of photosynthetic pigments, no photosynthetic activity was observed, as the F_V_/F_M_ values remained below 0.1 (Figure 6A,B). Similar to the protein content, more pigments were found in tissues cultured on solid media. Biochemical tests confirmed that a solid medium, under the conditions used in this study, is better suited for cultivating *Tussilago* callus. Therefore, the suspension cultures were not continued or further analyzed.

Since we were unable to find reference data on the relative amounts of photosynthetic pigments in *T. farfara* leaves, we measured the pigment content in the leaves of this plant grown in vitro (Figure 2A) to explain the unusually high relative Chl b content in the analyzed callus (Figure 6B). For comparison, we also examined the pigment content in the callus grown under light conditions, using identical light conditions for both the plant and the callus. The results are presented in Table 2.

Significant amounts of Chl a, Chl b, and carotenoids were found in the leaf tissues (Table 2). Notably, the Chl a/Chl b ratio, as well as the Chls/carotenoids ratio, was within the range observed in higher plants [23,24]. Leaf tissue contained, on average, about 32 and 21 times more Chl a and Chl b, respectively, and 14 times more carotenoids than the light-grown callus (Table 2). These differences may result, among other factors, from the type of tissue used for measurement. In the case of the callus, it was yellow-brown tissue with green regenerates, whereas the leaf blades collected for this study were completely green. Moreover, the relative pigment proportions were different. In the callus, there were relatively more carotenoids and Chl b than in leaf tissues. Nevertheless, the observed predominance of Chl b over Chl a in the dark-grown callus (Figure 6B; Table 2) was rather an exception. It is likely the result of Chl biosynthesis induced during the passage procedure, which was not performed in darkness, combined with Chl catabolism during the callus growth in the dark. Carotenoid synthesis can proceed in the absence of light [25].

### 2.3. Organogenesis

**Figure 7:**
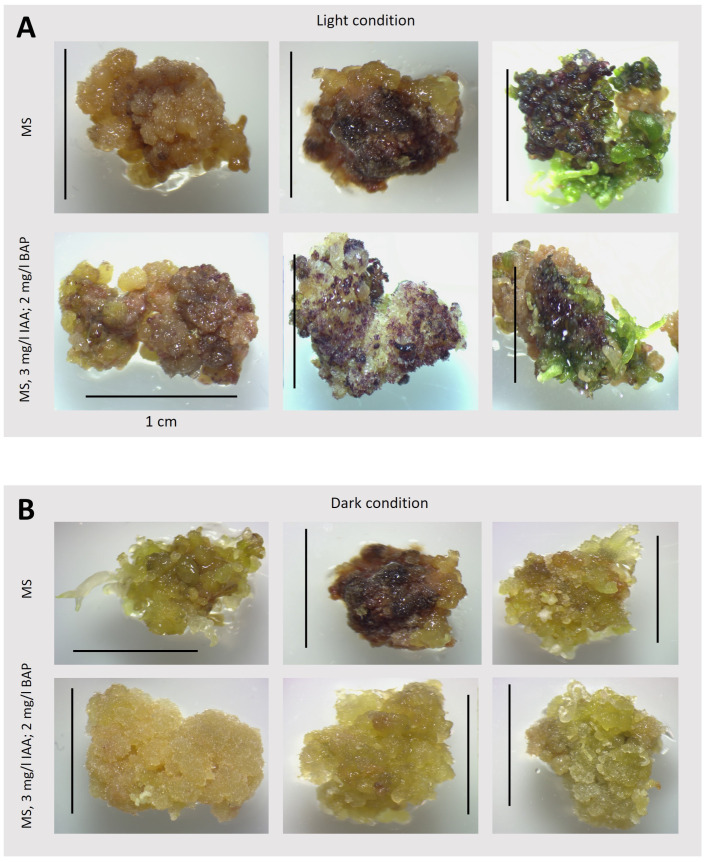
Organogenesis, color, and structure of an exemplary callus grown for 42 days on different media. (**A**): callus grown under light conditions 75 μmol photons m^−2^ s ^−1^; 14/10 (light/dark) photoperiod; (**B**): callus grown in dark conditions; Scale bar—1 cm.

Under the cultivation conditions of *T. farfara* in solid cultures that we studied, organogenesis was frequently observed. As shown by the results of callus induction (Figure 3 and Figure 4), the growth and efficiency of organogenesis depended on culture conditions, such as the presence or absence of light and the supplementation of appropriate hormones. Therefore, we examined the influence of light and darkness on the intensity of organogenesis using the medium identified as optimal for the intensive growth of the *T. farfara* callus in darkness (i.e., MS with 3 mg/L IAA and 2 mg/L BAP). For comparison, we also used the medium for the regeneration of *T. farfara* plants (i.e., MS), which was the source of leaf explants in our study (Figure 2A). The obtained results indicate that in the used in vitro conditions in the process of *T. farfara* organogenesis, light played an important and beneficial role, and a similar effect of light was observed in cultures of *Lilium usitatissimum* L., in which light clearly promoted organogenesis and its cyanogenic potential [26], as well as in cucumber cultures [27]. In addition to comparing callus color, we quantitatively analyzed the number and types of organs produced by the callus in these 42-day-old cultures (Figure 2E). The results are shown in Table 3 and Figure 7. Significant differences in callus morphology were observed, as well as variations in the number and color of regenerates depending on the culture conditions. Pieces of callus were usually inhomogeneous in color, with tints of various colors easily observable (Figure 7) and quantified in Table 3. The observed violet tints indicated the accumulation of anthocyanins [22].

The callus grown in the dark on the selected optimum medium (i.e., MS with IAA and BAP, Table 1) was yellow or slightly brown with a small amount of anthocyanins (Figure 7; Table 3). In contrast, the callus grown on a medium dedicated to plant regeneration (MS only) was noticeably darker. The callus grown under light conditions appeared completely different; it was light brown or slightly dark brown with a large amount of anthocyanins, especially when grown on medium with IAA and BAP. In this case, a larger amount of regenerates was also observed. While most regenerates on the callus grown in the dark were characterized as roots, in light conditions, most were leaves, especially on medium with only MS (Table 3, Figure 7). An interesting correlation with exposure to growth regulators is shown by regenerants that retain a greenish color in the absence of plant growth regulators in the medium and are deprived of green color (they are colorless) when exposed to the presence of IAA and BAP. A reduction in the number of albino spring barley microspores was obtained by replacing BAP with another cytokinin TDZ (thidiazuron) [28]. It is possible that such an effect could also be obtained in *T. farfara* cultures using this amine cytokinin, which may not terminate/interrupt the de-etiolation process but delays it, as indicated by the data obtained from the measurement of photosynthetic pigment activity. Symons and Reid [29] discuss the interactions between light and plant hormones and their role in mediating phenotypic change during de-etiolation. Recent molecular data provide evidence of interactions between the light and IAA/CK signaling pathways and it is possible that, in this aspect, the explanation of the discussed issue should be sought in the future.

### 2.4. Histological Callus Analysis

**Figure 8:**
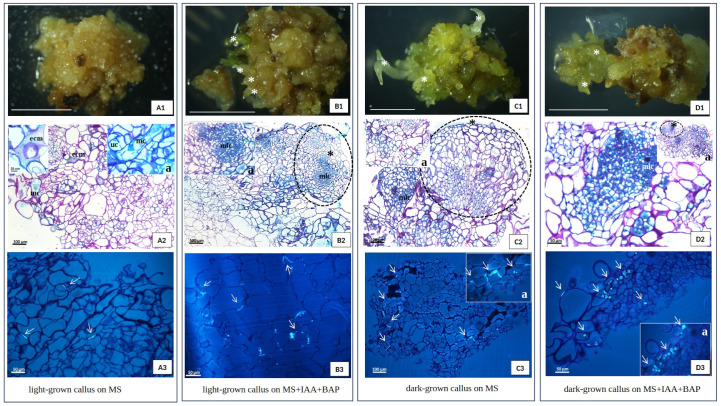
Histological analysis of *T. farfara* callus sampling 42 days after subculture. (**A**): light-grown callus on MS, (**B**): light-grown callus on MS + 3 mg/L IAA + 2 mg/L BAP, (**C**): dark-grown callus on MS, (**D**): dark-grown callus on MS + 3 mg/L IAA + 2 mg/L BAP; (**A2**,**B2**,**C2**,**D2**)—Pass and Naphtol Blue Back staining, (**A3**,**B3**,**C3**,**D3**)—aniline blue staining; arrows—callose deposition, asterisk—regenerants, ecm—extra cellular matrix, mlc—meristematic like cells, uc—unfinished cytokinesis, mc—multinucleated cell; Scale bar of (**A1**,**B1**,**C1**,**D1**)—0.5 cm; light intensity—75 μmol photons m^−2^ s ^−1^.

Histological analysis of 42-day-old callus samples grown on solid media (MS or MS with 3 mg/L IAA, 2 mg/L BAP, and 0.6% agar) selected for the highest morphogenetic potential, from dark and light conditions (75 μmol photons m^−2^ s ^−1^) (Figure 2E), confirmed previous macroscopic observations. Although the histological analysis mostly proved the organogenesis appearance (Figure 8(B1,C1,D1)) in each culture type, the symptoms of somatic embryogenesis could not be excluded, because they were noticeable in the form resembling proembryogenic structures, which is characteristic only for the light conditions. Similar structures were present in in vitro cultures of another member of the Asteraceae, *Taraxacum belorussicum*, which analogously stopped early in their development and did not develop into distinct somatic embryos [30]. The presence and distribution of the extracellular matrix (ECM) also appear to be light-dependent, with a larger quantity of it noticeable for the callus maintained in light compared to the one in darkness, and was mostly located on the edge of the callus (Figure 8(A2,D2)). Recent research shows that the ECM composed of proteins and polysaccharides and has a big influence on the functioning of cells, ensuring, among other things, effective cellular communication and protection [31]. Furthermore, it was shown that the ECM implies an active role in plant morphogenesis [31,32,33]. Under light conditions, starch was stored in the plastids, which were often located around the nucleus. The lack of light had a negative impact on the presence of starch in callus cells; it was found there sporadically. A common and characteristic feature of the callus from each experimental system was its high cytological diversity. The callus clusters consisted of large, irregularly shaped, and highly vacuolated cells, along with small isodiametric cells with a low vacuolization degree, dense cytoplasm, and very visible multi-nucleoli nuclei resembling the cells of meristematic character. Such cells were present both among the callus clumps and within adventitious shoots and roots (Figure 8). In the tested callus derived from each condition, cell differentiation toward the vascular tissue was not observed. Neither xylem nor phloem elements were present in analyzed samples, except in a few cases from light and dark conditions and PGR supplementation, where slight traces of individual/single protoxylem vessels were noticeable. It seems surprising that a long-term, stabilized callus lacks neovascularization, which ensures optimal transport of nutrients and is common in many other callus cultures [30,34,35]. However, the phenomenon of *T. farfara* is not an isolated one, as the stabilized non-morphogenic callus of kiwi (*Actinida arguta*) also completely lacks elements of vascular tissues, which is related to the specific level of auxins and cytokinins [34]. When looking for the causes of this phenomenon, one should take into account the supplementation of sugar and auxins, which, as confirmed by the literature data, are necessary for the neovascularization of the callus [36,37,38]. These factors were ensured in the studied coltsfoot callus cultures, so the reasons for the lack of induction of a sophisticated vascular network consisting of water-conducting vessels and assimilate-transporting sieve elements should be probably looked for in the species specificity and the selection of appropriate auxins.

In the histological images of all experimental systems of the studied *T. farfara* callus, the presence of multinucleated cells (Figure 8(A2)) and the presence of several nucleoli in large nuclei were clearly common (Figure 8(B2)), as well as the unfinished cytokinesis appearance (Figure 8(A2)). This phenomenon, following Häsler’s observations [39], can be attributed to the characteristics of cell cancer. The authors observed the loss of totipotency in callus cells, which then started to acquire some features (polynucleolation, nuclear invaginations, an incomplete cell wall, etc.) that are typical of neoplastic cells. Images of mitotic divisions are in fact very rare, which may suggest that the increase in calli biomass relies more on these types of atypical, partial, or complete cell divisions than on simple mitosis [39]. The observation revealed that even weak mitotic activity was correlated with callose distribution. Callose was deposited in different places and this varied localization was related to the conditions of the callus culture. In the dark-grown callus without PGRs, callose was present in the cell plate and was probably visible during cytokinesis (Figure 8(A3)) in much smaller amounts than in the callus originating from light and darkness on medium supplemented with PGRs (Figure 8(B3,C3,D3)). Light conditions and exposure to IAA and BAP resulted in two modes of callose deposition. In the walls of strongly vacuolated, large parenchymal cells, a thin polysaccharide deposit was observed, while in the walls of smaller cells, also of a parenchymal nature, callose deposits took the form of extremely thick plugs (Figure 8(B3,C3,D3)) that were so thick they refracted light in the microscope. Taking into account the multiple functions of callose and the fact that the examined callus cultures lack phloem elements, the presence of callose in this material translates, among other things, into mediating the separation of daughter cell nuclei as a transient component of the middle lamina during cytokinesis. Moreover, the controlled deposition of callose in plasmodesmata regulates simplistic transport in the callus, and the thick deposits of this polysaccharide can be explained by an enhanced response to abiotic stress. Under natural conditions, such a thickened structure, called a papilla, prevents the entry of pathogens during infection [40].

### 2.5. Photosynthetic Activity of Morphogenic Callus

**Figure 9:**
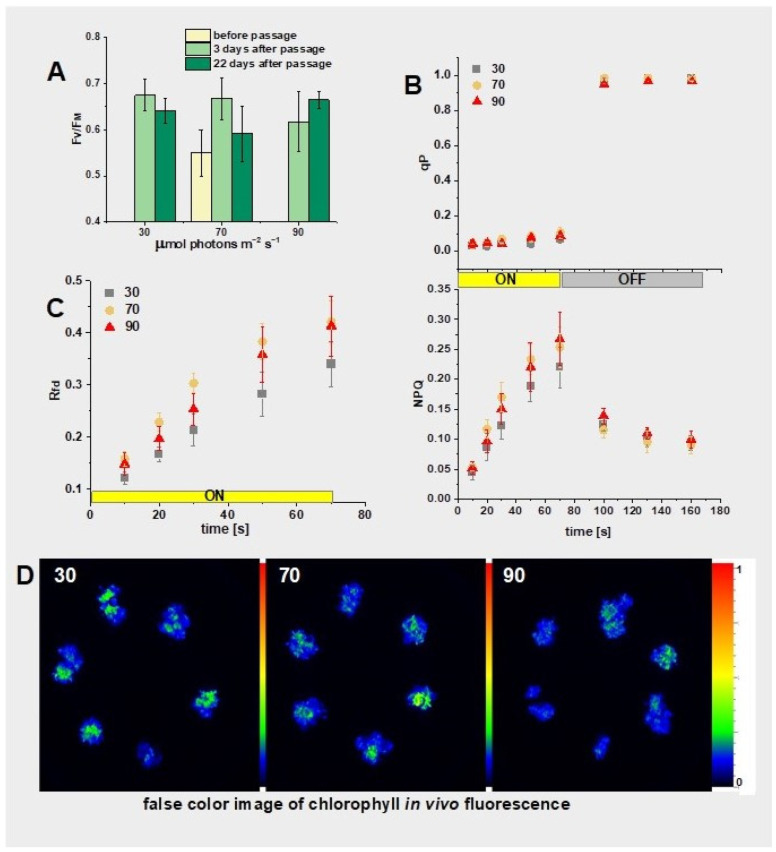
Photosynthetic parameters of the callus with adventitious shoots obtained from a chlorophyll in vivo fluorescence image. The culture was performed on MS, 3 mg/L IAA; 2 mg/L BAP under different light intensities (30, 70, and 90 μmol photons m^−2^ s^−1^; labeled as 30, 70, and 90, respectively). (**A**): F_V_/F_M_; (**B**): Coefficient of photochemical quenching (qP) and nonphotochemical quenching (NPQ) measured for 22 days after passage; (**C**): Vitality index (R_fd_) measured in parallel to qP and NPQ; (**A**–**C**) Average values and standard deviations are shown for n = 6; (**B**,**C**) The yellow and gray rectangles indicate turning on and off the actinic light (600 μmol photons m^−2^ s^−1^), respectively. (**D**): False color fluorescence image (F_M_) of a 28-day-old callus grown under different light intensities; see Section 3 for further explanations.

Finally, we studied the ability of the regenerated shoots and roots to grow under different light intensities. The dark-grown callus with developed adventitious shoots (Figure 2E) was transferred to white light (70 μmol m^−2^ s^−1^) for 6 days. Then, it was subcultured and cultivated under different white light intensities: 30, 70, and 90 μmol photons m^−2^ s^−1^ (Figure 2F) for 22 days. Light treatment led to the transformation of the tissue from yellowish to greenish. Photosynthetic activity was assessed using chlorophyll fluorescence imaging after 6 days under light before the passage. Then, it was assessed at 3 and 22 days after the passage. Samples were preadapted to darkness for 10 min each time (Figure 9). The average F_V_/F_M_ value, representing maximum PSII quantum yield [18], was the lowest for the callus before the passage, i.e., after 6 days of growth in light. It significantly increased in cultures grown under 30 and 70 μmol photons m^−2^ s^−1^ after 3 days of growth on fresh medium. However, this increase was less evident in the case of callus growth under 90 μmol photons m^−2^ s^−1^ (Figure 9A). In turn, only in this case was an increase in F_V_/F_M_ observed after 22 days of cultivation, while a decrease in this parameter was noted for cultivations at 30 and 70 μmol photons m^−2^ s^−1^. Nevertheless, even the highest observed F_V_/F_M_ is much lower than values characteristic for plants, which are usually around 0.83–0.86 [41]. This indicates that the photochemical PSII efficiency in a callus with adventitious shoots is lower than in plants. The impact of plant growth regulators on F_V_/F_M_ on the *N. tabacum* callus grown under the photoperiod has already been shown [42]. However, it needs to be remembered that this effect might be species-specific. It should also be taken into account that in our experiment, the callus with adventitious shoots was formed under dark conditions and then exposed to light, which induced chlorophyll biosynthesis and chloroplast biogenesis—a typical phenomenon for angiosperms [43,44]—resulting in greening of the tissues. At the same time, the process of biogenesis of the photosynthetic complexes took place, specifically PSII assembly. Not-well-assembled PSII, as well as an amount of chlorophyll not yet bound to the photosynthetic complexes, might be reflected by low F_V_/F_M_ values. An increase in F_V_/F_M_ to its maximal value during seedling de-etiolation has been already shown [45,46].

Further analysis of chlorophyll fluorescence signals provided more parameters characterizing photosynthetic activity, namely the coefficient of photochemical quenching (qP) and nonphotochemical quenching (NPQ), as shown in Figure 9B. The qP parameter was very low under the actinic light treatment (below 0.1). This parameter represents the fraction of open PSII centers, thus participating in the photosynthetic electron transport chain [47]. The obtained results indicate rapid saturation of this chain. Furthermore, a rapid opening of the PSII centers was observed after switching off the actinic light, demonstrating good PSII functioning during the light-to-dark transition. In line with this observation, switching on the actinic light induced an increase in the NPQ parameter, reflecting the increased dissipation of excess absorbed energy [48]. This means that light-harvesting antennae complexes are already assembled and functioning, although the maximal NPQ reached by the callus is very low when compared to leaves. Additionally, the empirical parameter, the vitality index (R_fd_), shows typical induction in light and reached slightly lower levels for the callus grown under the lowest light intensity (Figure 9C). Moreover, this parameter was always smaller than that reported for leaves [49].

**Figure 10:**
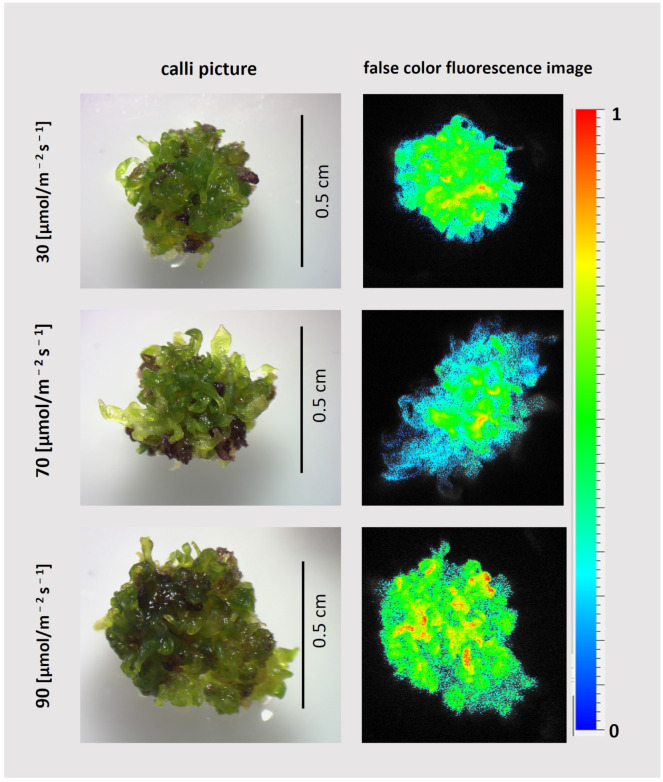
Sample close-up images of callus cultured for 22 days on MS, 3 mg/L IAA; 2 mg/L BAP under light with different intensities, as shown in the figure (**left side**), along with the corresponding false color chlorophyll fluorescence image, corresponding to F_M_ and induced with a saturating pulse of white light (**right side**); see Section 3 for details.

Chlorophyll fluorescence intensity imaged in vivo was unevenly distributed on the callus surface (Figure 9D). Close-up images revealed that the surfaces of all calli were covered with green and fluorescent adventitious shoots (Figure 10). The violet color visible among the shoots, slightly more intense in cultures treated with 90 μmol photons m^−2^ s^−1^ than observed under lower light intensities, reflects anthocyanin accumulation, as demonstrated in other research [20].

We showed that morphogenic callus in dark-grown cultures can develop photosynthetically active shoots within 6 days of light treatment. Moreover, these newly formed shoots exhibit traits of adaptation to different light intensities and can be transferred to higher or lower light intensities without harm.

## 3. Materials and Methods

### 3.1. Plant Material for Callus Induction

The plant material used for callus induction in this study was *T. farfara* (coltsfoot) plant from a sterile culture, which was regenerated from a callus (hereafter referred to as the ‘primary callus’) in our laboratory via the following procedure. First, a wild-growing *T. farfara* specimen [Figure 1] was collected (summer 2015) from meadows near the Campus of the 600th Anniversary of the Jagiellonian University in Kraków (50°01′44,396″ N 19°54′17,496″ E; Poland).

From this stage onwards, all work was carried out under sterile conditions. Fragments of blade leaves taken from the *T. farfara* plant were surface sterilized by immersion in 70% ethanol for 1 min, followed by immersion in 10% commercial bleach (ACE, Procter and Gamble DS Poland) with a few drops of Tween 20 for 20 min. They were then rinsed three times in sterile deionized water and cut into slices of approximately 15 to 20 mm. Such explants were placed on 90 mm Petri dishes containing Murashige and Skoog media [50] (MS, Duchefa, Haarlem, The Netherlands) supplemented with 3% sucrose (Chempur; Piekary Śląskie, Poland), 2 mg/L 4-amino-3,5,6-trichloropicolinic acid (Picloram, Duchefa, Haarlem, The Netherlands), and 2 mg/L BAP (Duchefa, Haarlem, The Netherlands). The pH of the medium was adjusted to 5.8 and supplemented with 0.6% agar (Roko, Spain) prior to autoclaving (20 min at 121 °C) according to [51]. The primary callus induction was carried out in the dark in a test chamber (Versatile Environmental Test Chamber, MLR 351H, Sanyo, Japan) at 23 °C. Callus cultures were subcultured monthly (four times) onto fresh medium.

For the regeneration of a plant in vitro, a piece of the callus (about 0.6 cm^3^) was placed in a 50 mL falcon tube with 15 mL of medium (MS medium, pH 5.8, supplemented with 0.1 mg/L BAP and 30 g/L sucrose, and solidified with 0.6% agar). The cultures were kept at 20 °C under white light illumination (75 μmol photons m^−2^ s ^−1^) and a 14/10 (light/dark) photoperiod for up to 10 weeks. The cultures of regenerated *T. farfara* plants were refreshed by transferring explants in the form of stems with 1 or 2 leaves onto fresh medium every 8 weeks. The cultivation conditions remained unchanged.

This culture was maintained for 5 years before being used as the starting material (leaf explants) for the callus production described in this study.

### 3.2. Callus Induction Protocol

Callus culture was induced on explants taken from the second and third leaves of 5-to-10-week-old regenerated *T. farfara* plants grown in sterile cultures (Figure 2A). Leaves cut in half crosswise or lengthwise were placed on Petri dishes with MS medium and 30 g/L sucrose, pH 5.8, and solidified with 0.6% agar. The medium was supplemented with different combinations of plant growth regulators from the following: 2–3 mg/L indoleacetic acid (IAA), 1–2 mg/L 2,4-dichlorophenoxyacetic acid (2,4-D), 2 mg/L naphthalene acetic acid (NAA), and 2–3 mg/L benzyl aminopurine (BAP). The specific hormone compositions are provided in the results section. The cultures were grown at 20 °C either under a cool white fluorescent lamp (75 μmol photons m^−2^ s^−1^) with a 14/10 (light/dark) photoperiod or in darkness. The observations were carried out after 14 and 28 days (Figure 2B).

After the callus induction (i.e., after 28 days), callus tissue was separated from explants as much as possible and placed on Petri dishes with the same medium as used for callus induction. Callus growth was carried out under the same experimental conditions as initiation. Every 4 weeks for two years, the callus was subcultured to the same fresh medium (Figure 2C).

### 3.3. Callus Growth

Callus cultures (Figure 2C) were transferred and subsequently grown in suspension (in 50 mL cell culture bottles; Figure 2(D1)) or on solid medium (on Petri dishes; Figure 2(D2)). The MS medium (pH 5.8) contained 30 g/L sucrose and growth regulators: 3 mg/L IAA and 2 mg/L BAP. For solid cultures, the medium was solidified with 0.6% agar. Both suspension and solid cultures were kept at 20 °C in darkness.

To study the light effect on callus culture, some Petri dishes were kept under illumination (75 μmol photons m^−2^ s^−1^) and a 14/10 (light/dark) photoperiod, while the rest were kept in the dark (Figure 2E). Finally, the effect of different light conditions on callus growth and organogenesis was studied under different light intensities: 30, 75, or 90 μmol photons m^−2^ s^−1^ (Figure 2F).

### 3.4. Callus Growth Kinetics

For callus growth kinetics, the fresh mass of callus clumps was examined (Figure 2(D1,D2)). Changes in callus mass on solid media were checked every 7 days and the culture conditions were not refreshed. For technical reasons, in liquid media, these measurements were taken twice: at the beginning of the experiment before inoculation into the medium and at the end of the experiment on the 42nd day of culture.

The values were taken for calculation of the Growth Index (GI, [14] and Relative Growth Rate (RGR, [15]) parameters using the following equations:

GI = (W_t_ − W_0_)/W_0_ (1)

RGR = (lnW_t_ − lnW_0_)/(t − t_0_) [mg/day] (2)

where: W_t_—the fresh weight of the callus at time t

W_0_—the initial fresh weight of the callus at the beginning of the treatment (t_0_) or at the beginning of the harvest interval

ln—denotes the natural logarithm

t—time (day of experiment)

t_0_—beginning of experiment or harvest interval

### 3.5. Biochemical Analysis

The callus (Figure 2(D1,D2,F)) was disintegrated by using grinding rods and garnet beads (quality: 50 pcs) in an Eppendorf tube using a mini homogenizer (Tissue mini grinder E0357, EURx, Gdańsk, Poland) kept on ice in the presence of an appropriate medium (depending on the biochemical analysis).

Protein analysis was performed in disintegrated tissue in the presence of 1 M NaOH and water (1:1). After centrifugation (5 min, 12,000 rpm, Eppendorf) protein concentrations were measured using the Lowry method [52].

Chlorophylls and carotenoids were extracted from disintegrated tissue using 80% acetone. After centrifugation (5 min, 12,000 rpm, Eppendorf), the concentration of chlorophylls and carotenoids in the extract was determined with a spectrophotometer (Jasco 870, Easton, MD 21601, USA) and calculated using the Lichtenthaler method [53].

The content of anthocyanin pigments was determined using the differential spectrophotometric method of Giusti and Wrolstad [54]. Anthocyanin pigments from the callus were extracted using an 80% ethanol solution acidified by 0.1 N hydrochloric acid. The extract was then mixed with a buffer of pH 1 and 4.5 and the absorbance was measured at a wavelength of 530 and 800 nm (Metertek SP-930 spectrophotometer, Taiwan).

### 3.6. Observation of Callus Tissue

Calli observation and photographic documentation was made using a stereo microscope (SK Series Microscope, OPTA-TECH, Warszawa, Poland) equipped with an HDMI series camera and OPTAViewIS (ver. 4.3.0.6001) directly on Petri dishes. Based on observations and photographic documentation made using camera software, an analysis of the callus morphology and organogenesis was carried out.

### 3.7. Histological Analysis of Callus Formation

Calli samples (Figure 2E) from each kind of media (MS or MS with 3 mg/L IAA, 2 mg/L BAP, and 0.6% agar) and different conditions (dark or light: 75 μmol photons m^−2^ s ^−1^) after 42 days of culture were fixed in a mixture of 5% glutaraldehyde and phosphate buffer, pH 7.2 (Sigma, St. Louis, MO, USA), at 5 °C overnight. Next, the samples were washed four times in 0.1 M phosphate buffer (pH 7.2), dehydrated in an ethanol series (10%, 30%, 50%, 70%, and 96%; 15 min each), and kept overnight in absolute ethanol. The fixed calli were subsequently embedded in Technovit 7100 (Heraeus Kulzer, South Bend, IN, USA) synthetic resin. Plant material histology block slices (7 μm of thickness) were obtained using a rotational microtome—Microm HM 355 S I Microtome (MICROM International GmbH, Walldorf, Germany).

The sections were stained with 0.1% toluidine blue O, naphthol blue black for proteins detection [30], periodic acid-Schiff’s (PAS) reagent for water-insoluble polysaccharides identification [55], modified by [56]), and aniline blue for callose [30], and mounted in histological medium (Enthellan; Merck Millipore, Burlington, MA, USA).

Observations and documentation were performed using a Nikon Eclipse E400 light microscope. The sections treated with aniline blue were analyzed under UV light using a Nikon Eclipse E400 microscope (Nikon Corporation, Tokyo, Japan) with an Epi-FL Filter Block N UV-2A (EX330–380, DM400, BA420). Photographic documentation was made with a digital camera and NIS-Elements D software ver 4.0.

### 3.8. Chlorophyll In Vivo Fluorescence Measurements

Chlorophyll in vivo fluorescence methods were used for analysis of photosynthetic activity [57,58]. Fv/Fm measurements were performed using a Pocked PEA Chlorophyll Fluorimeter (Hansatech Instruments, Pentney, UK) according to a standard protocol. Before the measurement, callus fragments (approx. 100 mg) (Figure 2(D1,D2)) were dark-adapted for 15 min.

Chlorophyll fluorescence imaging of callus samples (Figure 2F) on Petri dishes (MS with 3 mg/L IAA, 2 mg/L BAP, and 0.6% agar) grown under different light conditions (30, 75, and 90 μmol photons m^−2^ s ^−1^) was performed using a pulse-modulated Open Fluor-Cam FC 800-O/1010 fluorimeter and analyzed using FluorCam7 software ver 1.2.5.24 (PSI, Drasov, Czech Republic). The standard ‘F_V_/F_M_’ and ‘QuenchingAct2’ protocols were used. Samples were pre-incubated in darkness for 10 min immediately before measurement. Image and setting optimization were performed before darkening. The intensity of the saturating pulse was set at 3700 μmol photons m^−2^ s^−1^ and that of the actinic light was 550–600 μmol photons m^−2^ s^−1^. The following parameters were calculated from the quenching analysis: NPQ (nonphotochemical quenching), qP (coefficient of photochemical quenching, which estimates the fraction of open PSII reaction centers), and R_fd_ (fluorescence decline ratio; an empiric parameter used to assess plant vitality). The average values for 6 repetitions, along with respective standard deviations, were calculated.

## 4. Conclusions

The efficient protocol for the induction, stabilization, and continuous growth of callus from *T. farfara* in vitro regenerants has been established. The viable callus achieved via secondary induction was maintained continuously for two years. It is recommended that the *Tussilago* callus be grown in darkness; light reduces the growth rate. In the dark, the callus maintains a constant growth rate, which may be related to the anergization phenomenon. Under these conditions, higher mitotic activity was observed, as confirmed by histological images showing callose presence in plate cells. The appearance of the ECM (extracellular matrix) and symptoms of somatic embryogenesis, based on histochemical analysis, indicate a high morphogenetic potential of this long-term callus. Light enhances organogenesis.

We have also shown that, under experimental conditions, indirect organogenesis is achievable, even with a growth protocol designed for callus tissue.

Establishing a long-term callus culture protocol is an important tool for biotechnological sources of metabolites. Our research is also significant in the context of climate change, when climate warming may adversely affect the development cycle of this early spring species. The potential interference in the natural habitat of *T. farfara* caused by climate change and the shortening of the spring period may also pose a threat to coltsfoot. Developing in vitro cultivation methods can help safeguard the native gene pool, and open the possibility for future reintroduction of endangered species to the natural habitat.

Finally, we demonstrate that the regenerated callus has low photosynthetic activity compared to leaves, but this activity is not dependent on light conditions. Additionally, chlorophyll fluorescence induction and relaxation were similar to that of plants, albeit weaker.

## Figures and Tables

**Table 1:** Efficiency of callus induction on solid cultures with MS medium and different plant growth regulators under dark or light conditions (75 μmol photons m^−2^ s^−1^; 14/10 (light/dark) photoperiod). Plus signs (+, ++, +++) indicate the conventional scale of callus growth after initiation, while a minus sign (−) indicates no initiation. (IAA—indole-3-acetic acid; 2,4-D—2,4-dichlorophenoxyacetic acid; NAA—naphthalene acetic acid; BAP—benzyl aminopurine.

Growth Conditions	Hormone Composition in MS Medium			Callus Initiation [+++/−]
Light	2,4-D (1 mg/L)	NAA (2 mg/L)	BAP (2 mg/L)	+
	2,4-D (1.5 mg/L)	NAA (2 mg/L)	BAP (2 mg/L)	+
	2,4-D (2 mg/L)	NAA (2 mg/L)	BAP (2 mg/L)	+
	IAA (3 mg/L)	-	BAP (2 mg/L)	++
Dark	2,4-D (1 mg/L)	NAA (2 mg/L)	BAP (2 mg/L)	+
	2,4-D (2 mg/L)	-	BAP (3 mg/L)	+/−
	IAA (3 mg/L)	-	BAP (2 mg/L)	+++
	IAA (2 mg/L)	-	BAP (2 mg/L)	++

**Table 2:** Photosynthetic pigment content in *T. farfara* leaves from plants grown in vitro and in callus from solid cultures grown under light and dark conditions. Light conditions were identical for both the callus and the plants: 70 μmol photons m^−2^ s ^−1^ with a 14/10 (light/dark) photoperiod. Growth medium for plants: MS; growth medium for callus: MS with 3 mg/L IAA and 2 mg/L BAP. n = 3, where n represents individual pieces of callus or leaves.

Plant Material	Chl a [ng/mg]	Chl b [ng/mg]	Carotenoids [ng/mg]	Chl a/ Chl b	Chl/ Carotenoids
Light conditions					
leaves	1095.3 ± 148.6	345.5 ± 45.0	244.3 ± 32.1	3.17 ± 0.04	5.90 ± 0.22
callus	33.5 ± 9.6	16.3 ± 1.96	17.06 ± 4.13	2.03 ± 0.40	2.93 ± 0.05
Dark conditions (*)					
callus	0.78 ± 0.56	1.48 ± 0.88	3.51 ± 0.58	0.50 ± 0.08	0.66 ± 0.40

* the data are also shown in Figure 6B.

**Table 3:** Characteristics of the callus and organogenesis in 42-day-old solid cultures (R—root; L—leaf; S—shoots). Light conditions: 75 μmol photons m^−2^ s^−1^; 14/10 (light/dark) photoperiod.

Growth Conditions	Medium	Callus Color and Tint with Another Color [%]	Calli with Organogenesis		White Organs [Number]			Green Organs [Number]		
			[%]	[Number]	R	L	S	R	L	S
Light	MS	light brown tinted on dark brown (10.9) on violet (13)	21.8	10	2	9	2	0	17	3
	MS + IAA (3 mg/L), BAP (2 mg/L)	light brown tinted on dark brown (26.1), on yellow (26.1) on violet (45)	23.9	11	14	14	8	0	1	0
Dark	MS	brown tinted on yellow (13)	19.4	9	11	4	3	0	0	0
	MS + IAA (3 mg/L), BAP (2 mg/L)	light yellow tinted on brown (15.2) on violet (17.4)	17.4	8	17	2	0	0	0	0

## Data Availability

The original contributions presented in this study are included in the article, further inquiries can be directed to the corresponding author.
